# Modeling and Optimizing the Composite Prepreg Tape Winding Process Based on Grey Relational Analysis Coupled with BP Neural Network and Bat Algorithm

**DOI:** 10.1186/s11671-019-3118-4

**Published:** 2019-08-28

**Authors:** Bo Deng, Yaoyao Shi

**Affiliations:** The Key Laboratory of Contemporary Design and Integrated Manufacturing Technology, Ministry of Education, Northwestern Polytechnical University, Xi’an, 710072 China

**Keywords:** Composite tape winding process, Tensile strength, Void content, Backpropagation neural network, Bat algorithm

## Abstract

**Abstract:**

As a significant way to manufacture revolving body composite, the composite prepreg tape winding technology is widely applied to the domain of aerospace motor manufacture. Processing parameters, including heating temperature, tape tension, roller pressure, and winding velocity, have considerable effects on the void content and tensile strength of winding products. This paper was devoted to studying the influence of process parameters on the performances of winding products including both void content and tensile strength and trying to provide the optimal parameters combination for the objectives of lower void content and higher tensile strength. In the experiments, tensile strength and void content were selected as the mechanical property and physical performance of winding products to be tested, respectively. An integrated approach by uniting the Grey relational analysis, backpropagation neural network, and bat algorithm was presented to search the optimal technology parameters for composite tape winding process. Then, the composite tape winding process model was provided by backpropagation neural network utilizing the results of Grey relational analysis. According to the bat algorithm, the optimal parameter combination was heating temperature with 73.8 °C, tape tension with 291.2 N, roller pressure with 1804.1 N, and winding velocity with 9.1 rpm. The value of tensile strength increased from 1215.31 to 1329.62 MPa. Meanwhile, the value of void content decreased from 0.15 to 0.137%. At last, the developed method was verified to be useful for optimizing the composite tape winding process.

**Graphical Abstract:**

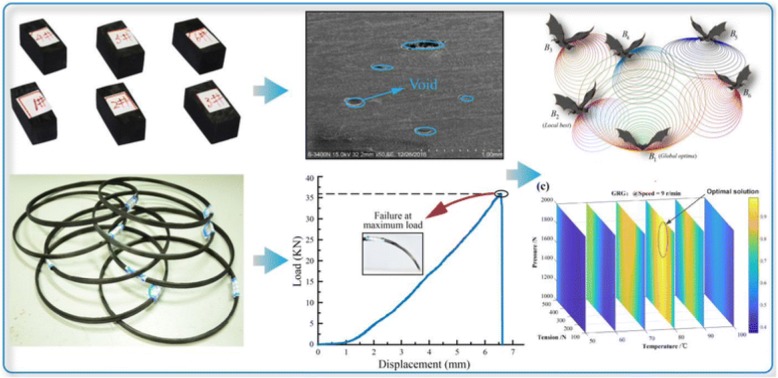

## Background

Advanced composites have made great achievements in the field of military, aerospace, industry, and civil application for the advantages of high specific intensity and specific rigidity. As a significant way to manufacture rotary composite parts, the composite prepreg tape winding technology is widely used to fabricate the aerospace products [1–4], such as ablation resistance parts, rocket motor nozzle, some high-temperature resistance components, and rocket launch tube. Therefore, composite winding products play an important role in the development of national defense and aerospace industry. In the composite tape winding process, four technological parameters including tape tension, heating temperature, roller pressure, and winding velocity have major impact on the composite winding products [4–7]. However, as a representative form of product defect, an excess of voids will markedly reduce the compactness and strength of final winding products. Meanwhile, for the composite manufacturing process, product strength is an important item which needs to be considered. Therefore, to research the composite winding process more comprehensively, not only physical performance but also mechanical properties of winding products should be given a good attention at the same time.

Recent years, composite prepreg tape winding technology has made a huge contribution for the aerospace industry. In the manufacturing field of high-strength rotational composite, especially for rocket motor nozzles, the tape winding process shows huge advantages and vitalities, such as high efficiency and high reliability. Currently, a large number of scholars in the world have carried out a lot of research about the composite tape winding process. Firstly, modeling the composite tape winding process is beneficial for researchers to comprehend the entire technological process from a macro level. The thermodynamic modeling of composite tape winding process proposed by Tannous et al. [8] showed that composite tape winding process could be carried out with a small tape tension in the event that the high frictional contact existed between the tape and compaction roller. However, during the filament winding process, the fiber bundle, more or less, will suffer damage on different degrees due to the tension and extrusion stresses. According to Akkus and Garip’s research [9], the damage of carbon fiber increases with the increasing of pretension force on the carbon fiber during the tape winding process. Therefore, to obtain the better performance of composite, the defects and strength of composite winding products should be lucubrated. Cui et al. [10] studied the influence rule of the winding tension on the appearance and performance of bearing composites using the T300/epoxy prepreg tape. Similarly, Okuya et al. [11] enhanced the bonding strength of winding products by co-curing the carbon fiber-reinforced plastics strand with end tabs. Then, the modified method was employed to two kinds of carbon fiber-reinforced plastics strands and generated the responsible tensile strength. Last but not least, the performances of composite winding products are intensively subjected to the four process parameters. A preferable winding parameters combination will improve both physical and mechanical performance for the composite winding products. As in Nayani Kishore Nath’s [12] article, the optimum set of composite tape winding technological parameter were found and used to improve the performances of throat back up liners based on robust design method. Besides that, Yu et al. [13] provided a composite prepreg tape winding technology theoretical model which could be applied to selecting optimal parameters and prominently enhance the interlaminar shear strength of winding products.

In summary, a good deal of the literatures concentrated on the theoretical simulation, process control and technological optimizing on the composite prepreg tape winding technology. We can see that modeling and optimizing of the composite tape winding process has attracted many scholars to study. Nevertheless, for optimizing of the composite tape winding process, the current researches are often about optimizing for one target or multiple targets of the same type. In other words, the evaluation standard for the performances of composite tape winding products should be more comprehensive. Researches show that a relative effective process parameter combination will have a great contribution to the composite tape winding manufacturing. Therefore, in this optimizing process of the composite tape winding technology, both physical and mechanical performance of winding products should be considered simultaneously instead of just only one of them. In view of this, void content was selected to measure the physical performance of winding products in this study process. At the same time, tensile strength was chosen as the test index for the mechanical properties of winding products. In the paper, an integrated methodology combining Grey relational analysis (GRA), backpropagation neural network (BPNN), and bat algorithm (BA) would be established to search the optimal technology parameters of composite tape winding process. The authors wish the study will be beneficial for the composite prepreg tape winding manufacturing technology and provide a better performance for the composite winding products.

## Experiment Procedure and Results

**Fig. 1:**
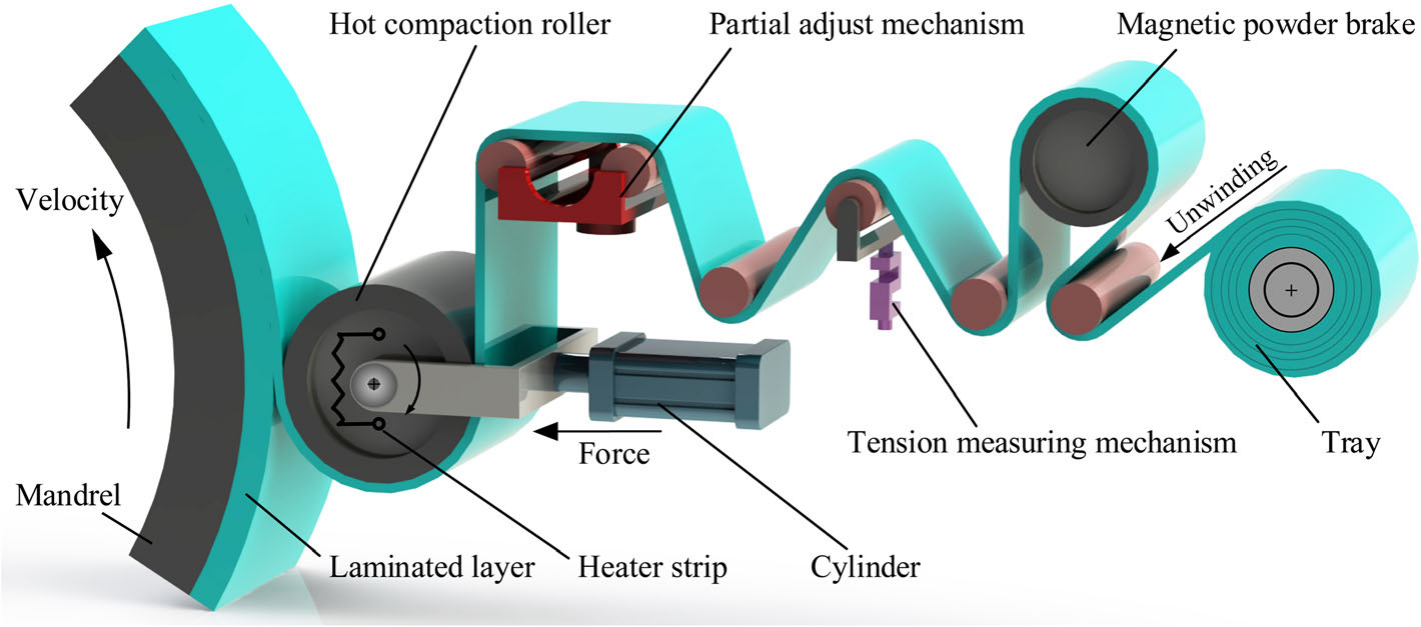
Three-dimensional diagram of composites prepreg tape winding process

The composite prepreg tape winding technology can be summarized as the hoop winding process prepreg tapes aiming at fabricating the revolving body composite. At the beginning, the prepreg tapes need to be heated to an appropriate temperature by the electric heating device which is located inside of the compaction roller. Meanwhile, magnetic powder brakes are utilized to guarantee that the prepreg tape keeps a certain constant tension. Simultaneously, the compaction roller pressure provided by the air cylinder is applied to the winding tape. Subsequently, the mandrel, following the spindle’s turning, rotates at an invariable speed. Then, the unwinding tapes are continuously winded to the outer layers of the mandrel. Figure 1 shows a three-dimensional diagram of the composites prepreg tape winding process. From the picture, we can see a tension measuring mechanism has been applied to ensure the correct tape tension. In order to reduce the product defects, a high-precision partial adjust mechanism is utilized to keep the proper winding trajectory. In the winding process, the heating temperature is conducive to bring down the resin viscosity and improve the interlaminar contact degree. In addition, the positive force produced by compaction roller will be applied on the prepreg tape. As the roller pressure squeezes out the bubbles among the interlaminar contact interfaces, the prepreg tape and laminated layers will not only contact intimately but also has a lower void content. Then, a suitable tape tension can greatly enhance the tensile strength for winding products. Last but not least, the mandrel velocity will have a major impact on the properties of winding products, for example, compactibility and uniformity. Therefore, both the mechanical and physical performances of winding products are deeply affected by the above four process parameters.

### Experiment Design and Sample Preparation

During the experiments, four critical process parameters of the composite tape winding technology including heating temperature, tape tension, roller pressure, and winding velocity were chosen as the design variables. At the same time, two key performances characterizations for tape winding products, tensile strength and void content, were selected as the evaluation indexes. And, more remarkably, the interval of each process parameter was designed on the basis of actual processing requirements. Therewith, the experiment was designed according to a four-factor and three-level Box–Behnken design (BBD) based on response surface theory. The experimental level of four key process parameters can be seen in Table 1.

**Table 1:** Level of process parameters

Experimental parameters	Symbol	Units	Level of experimental parameters		
			Level 1	Level 2	Level 3
Temperature	*T*	°C	50	75	100
Tension	*F*	N	100	300	500
Pressure	*P*	N	1000	1500	2000
Speed	*V*	rpm	5	10	15

**Fig. 2:**
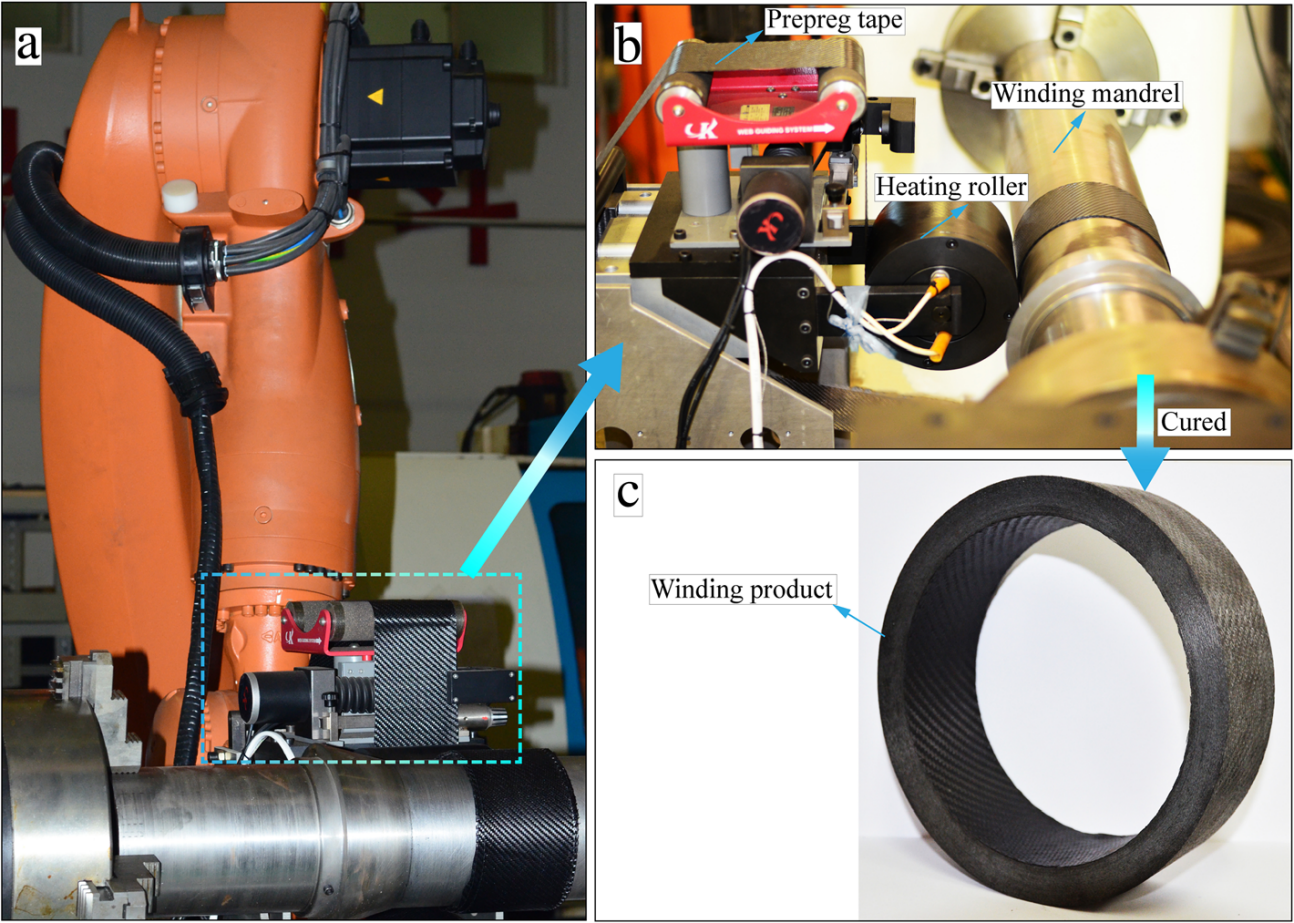
Automate tape winding KUKA robot (XGD-1200). **a** Mechanical arm of XGD-1200. **b** Tape winding head. **c** Cured winding product

In the experimental process, the composite prepreg tape, fabricated by Gloway Composite Materials Co., Ltd., was composed of carbon fiber and epoxy resin. The carbon fiber was T-300 provided by Toray Industries, Inc., and the epoxy resin matrix was YH-69 coming from Wuxi Resin Factory of Bluestar New Chemical Materials Co., Ltd. Furthermore, the fiber volume content of the composite prepreg tape was near 55 ± 2%. The width and thickness of the prepreg tape were 80 mm and 0.25 mm, respectively. The structure of composite prepreg tape was plain weave with the braided angles of 0°/90°. All the experiments were carried out on the Automate Tape Winding KUKA Robot (XGD-1200). This proprietary CNC equipment was designed and manufactured by Northwestern Polytechnical University, China, as shown in Fig. 2. Meanwhile, the XGD-1200 can be also applied to the composite prepreg tape placement process. The machine body is a horizontal structure with a full closed-loop control system. The size of the machinable parts (including the core mold) is less than 1500 mm in length and 50~ 1000 mm in rotary diameter. In addition, the winding mechanism uses the six-axis robot KR 180 R2500 extra fabricated by KUKA Aktiengesellschaft, Germany. The robot has a repeat accuracy of ± 0.06 mm and a working radius of 2500 mm. Furthermore, the technical parameters of tape winding process including winding temperature, roller pressure, and tape tension are controlled by SIMATIC S7-1200 PLC.

The winding specimens were manufactured with a manner of hoop winding. The ambient temperature and relative humidity of these experiments were 20 ± 2 °C and 25 ± 2%, respectively. The size of the compaction roller was 160 mm in external diameter and 150 mm in width with a material of 45 steel. The winding mandrel was a 45 steel cylinder which was 150 mm in external diameter and 1200 mm in length. A high-precision online rectifying deflection system was employed to ensure proper winding trajectory for decreasing the product defects. Ultimately, the winding products produced by multilayer prepreg tape were cured by autoclave provided by TEDA Industrial Equipment Co., Ltd. (Tianjin, China). During the cured process, the heating rate was kept at 2.5 °C/min. It is worth noting that the curing temperature needs to be kept for 150 min when the temperature rose from room temperature to 150 °C. Meanwhile, the curing pressure stayed around 0.15 MPa.

### Measurement Method

#### Tensile Strength

**Fig. 3:**
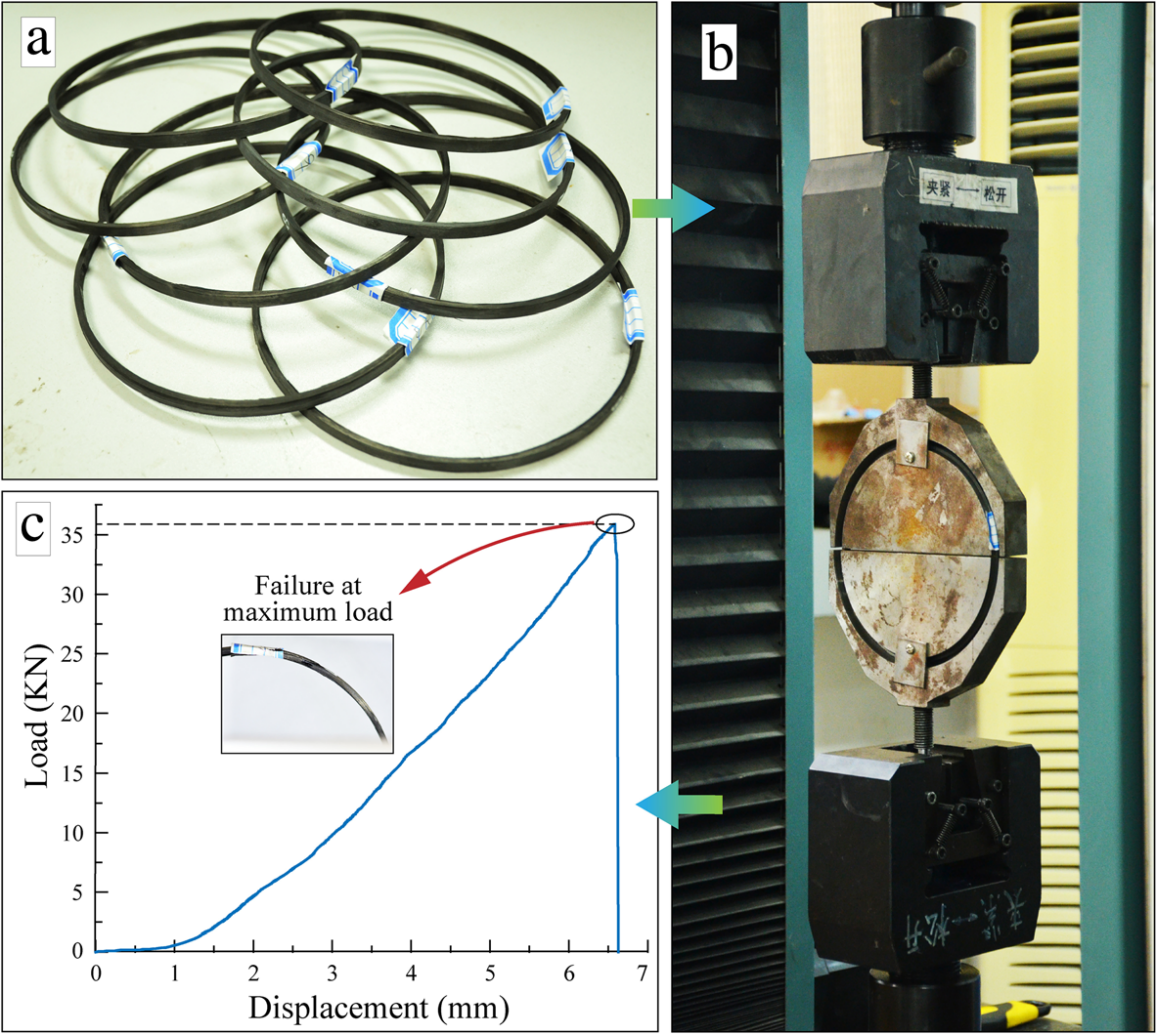
Experiment test samples machining and tensile strength testing process. **a** Standard testing ring. **b** Electronic universal testing machine DDL100. **c** Force- displacement tensile curve

The tensile strength measurement for winding specimens was proceeded based on the GB/T 1458-2008 standard [14]. At first, the standard testing ring, as shown in Fig. 3a, was obtained by mechanically cutting the cures winding specimen along the tape winding direction. Then, the STRs having a dimension of 150 ± 0.2 mm in inner diameter, 6 ± 0.2 mm in width, and 3 ± 0.1 mm in thickness would be utilized to test the strength. At last, the tensile strength tests were carried out with the help of electronic universal testing machine DDL100 (Fig. 3b) produced by Changchun Research Institute for Mechanical Science Co., Ltd. Meanwhile, the force-displacement tensile curve was provided in Fig. 3c. Then, the computational formula for the tensile strength of the fiber-reinforced composite can be described as

1 $$ {\sigma}_t=\frac{F_b}{2b\cdot h} $$

where *σ*_*t*_ denotes the tensile strength *T*_*S*_, *F*_*b*_ means the maximum load, and *b* and *h* are the width and thickness of specimens for tests, respectively.

#### Void Content

**Fig. 4:**
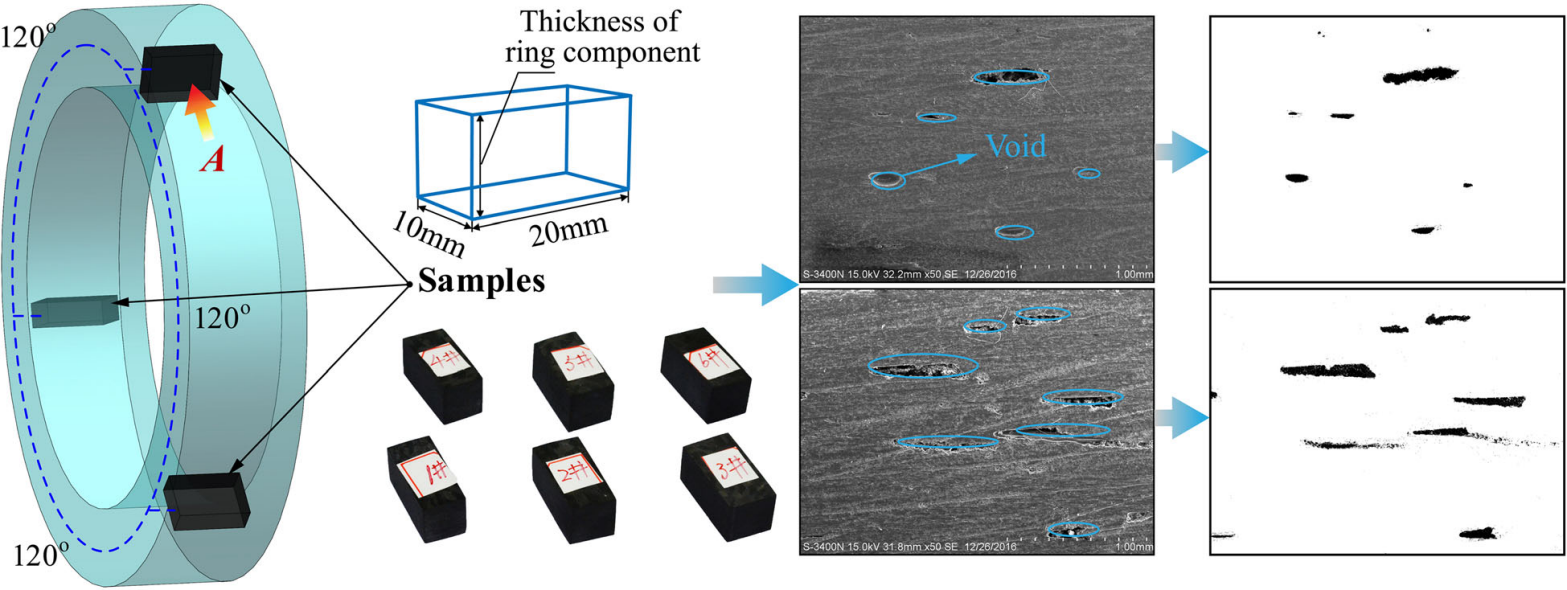
Process of measuring void content for composite tape winding products

Figure 4 gives the simplified schematic of how the test samples were obtained from a multi-layered cured winding product. The void content measurement for winding specimens was processed based on the GB/T 3365-2008 standard [15]. The dimension of the test sample was 20 mm in length, 10 mm in width, and thickness of ring component in height. Three samples were cut out from three cubical equidistant locations along the toroidal direction of winding component (Fig. 4). Whereafter, rough polishing for winding samples was carried out on the surface grinding machine in horizontal HZ-800/2CK produced by Hangzhou Hangji Machine Tool Co., Ltd. Then, corresponding polishing paste and cloth were employed to fine shine the experimental samples. For fiber reinforcement composite, micrography is a significant method to measure the void content of composite winding products [16]. Finally, the scanning electron microscope HITACHI S-3400 provided by Hitachi High-Technologies (Shanghai) Co., Ltd. was used to take photographs from the direction of ***A*** (red italics in Fig. 4). Then, post-processing including graying and binarizing for the micrographs was implemented in MATLAB version R2017a. Then, the computational formula for the void content of fiber-reinforced composite can be described as the ratio of all the voids acreage to the entire cross-sectional area, i.e.,

2 $$ {V}_C=\frac{A_{\mathrm{void}}}{A_{\mathrm{total}}}\times 100\% $$

where *V*_*C*_ denotes the void content of winding samples, and *A*_viod_ and *A*_total_ are the area of all voids and total cross-sectional acreage of the testing sample, respectively.

### Experiment Results

The testing data results including tensile strength and void content are pulled together as shown in Table 2. In the table, *T* denotes the heating temperature, *F* denotes the tape tension, *P* denotes the roller pressure, and *V* denotes the winding velocity. For tensile strength, the maximum and minimum values are 1215.31 and 783.26, respectively. For void content, the maximum and minimum values are 2.19 and 0.13, respectively.

**Table 2:** Experiment and measurement results

No.	Process parameters				Tensile strength (MPa)	S/N ratio	Void content (%)	S/N ratio
	*T*	*F*	*P*	*V*				
1	75	300	1000	15	896.92	59.0551	1.64	− 4.2969
2	50	300	2000	10	942.36	59.4843	1.19	− 1.5109
3	100	300	2000	10	1012.24	60.1057	0.25	12.0412
4	75	500	1500	15	951.33	59.5666	1.26	− 2.0074
5	75	300	1500	10	1210.68	61.6606	0.13	17.7211
6	75	300	2000	5	1123.47	61.0112	0.35	9.1186
7	100	500	1500	10	1062.27	60.5247	0.73	2.7335
8	50	500	1500	10	920.61	59.2815	1.45	− 3.2274
9	75	300	1500	10	1212.53	61.6739	0.15	16.4782
10	75	300	1500	10	1209.32	61.6508	0.17	15.3910
11	75	500	1000	10	1029.13	60.2494	1.39	− 2.8603
12	75	100	1500	5	967.14	59.7098	0.95	0.4455
13	75	500	2000	10	1018.74	60.1613	0.71	2.9748
14	100	100	1500	10	951.72	59.5702	0.87	1.2096
15	75	100	2000	10	866.58	58.7562	0.59	4.5830
16	75	300	1500	10	1213.24	61.6789	0.14	17.0774
17	75	300	1500	10	1215.31	61.6937	0.15	16.4782
18	100	300	1500	15	1130.29	61.0638	0.28	11.0568
19	50	100	1500	10	823.21	58.3102	0.79	2.0475
20	75	100	1500	15	876.68	58.8568	0.38	8.4043
21	75	100	1000	10	846.43	58.5518	1.68	− 4.5062
22	50	300	1500	5	1116.38	60.9562	0.31	10.1728
23	50	300	1500	15	783.26	57.8781	2.12	− 6.5267
24	75	300	2000	15	911.07	59.1910	1.16	− 1.2892
25	100	300	1500	5	1134.81	61.0985	0.27	11.3727
26	100	300	1000	10	997.85	59.9813	1.06	− 0.5061
27	75	500	1500	5	1053.11	60.4495	0.73	2.7335
28	50	300	1000	10	921.39	59.2889	2.19	− 6.8089
29	75	300	1000	5	1106.54	60.8793	0.79	2.0475

## Multi-objective Optimization Method

The composite prepreg tape winding technology is a multi-factor and multi-object process. In order to optimize the parameters of composite tape winding process, this paper provided an innovative solution which combined three optimization technologies including the Grey relational analysis (GRA), backpropagation neural network (BPNN), and bat algorithm (BA). First of all, the GRA is widely employed to obtain the approximation ratio among the data series in virtue of Grey relational grade (GRG). Meanwhile, the discrete sequences produced by GRA will be used to carry out the correlation analysis such as multiple factors, processing indetermination, and discrete data. At the same time, the backpropagation neural network (BPNN) is an artificial learning algorithm for multilayer neural networks and has a strong retrieval ability for functions. Due to the advantage of strong nonlinear interpolation ability, BPNN has been effectively used to find the interrelation between the process parameters and two performance characterizations of winding products in this study. At last, the bat algorithm, a patent evolutionary algorithm inspired by microbats’ echolocation behavior, can be usefully utilized to find the best process parameters combination quickly and reliably. Compared with other algorithms, bat algorithm has more superior accuracy and efficiency and does not have the need to adjust many parameters of the algorithm.

In the paper, Grey relational analysis, backpropagation neural network, and bat algorithm are integrated as a GRA-BPNN-BA method to model and optimize the complex multi-objective composite tape winding process. In order to improve efficiency and simplify the counting process, we choose to compute the signal-to-noise ratio (S/N) ratio of data sequence in the first place. The S/N ratio is defined as the ratio of signal power to the noise power, often expressed in decibels. In the composite tape winding process, a large value of tensile strength is expected, while the lower value of void content, the preferable performance of the winding product. Thus, the S/N ratio of the tensile strength and void content can be given as

3 $$ \eta =-10{\log}_{10}\left(\frac{1}{N}\sum \limits_{i=1}^N\frac{1}{T_{Si}^2}\right)\kern1em \left(1,2,\dots, N\right) $$

4 $$ \eta =-10{\log}_{10}\left(\frac{1}{N}\sum \limits_{i=1}^N{V}_{Ci}^2\right)\kern1em \left(1,2,\dots, N\right) $$

where *T*_*Si*_ and *V*_*Ci*_ represent the values of tensile strength and void content for the *i*th result in *N* tests, respectively; *η*_*TS*_ and *η*_*VC*_ are the corresponding S/N ratio value of the tensile strength and void content, respectively.

The results of S/N ratio are displayed in Table 2. For tensile strength, the maximum and minimum S/N ratio values are 61.6937 and 57.8781, respectively. For void content, the maximum and minimum S/N ratio values are 17.7211 and − 6.8089, respectively. At the beginning, the experiments for tensile strength and void content of winding products were carried out respectively. The experimental data should be carefully recorded and conformed to the corresponding test group. Thereafter, the GRA method was employed to describe the influence degree of technological parameters on the tensile strength or void content of winding products. Then, the BPNN method was applied to build the nonlinear mapping relations between technological parameters and GRG generated by the GRA. Finally, the BA method would be used to provide the optimal solution after a finite number of iterations. To verify the accuracy of the optimizing method, some validation tests were necessary to be carried out at the last step.

## Optimization Process and Discussion

### Grey Relational Analysis

The idea of the Grey theory is produced by the combination of multi-intelligence theory such as system theory, space theory, and control theory. Grey systems method can be applied to cope with numerous ambiguities caused by imprecise human decision-making. Even though the number of data is small or the variability of factors is great, the Grey system theory also can produce the satisfactory results [17, 18]. Through proper processing and regularization of data aggregation, Grey relational analysis can reveal the uncertain relationship between one major factor and all other factors in the system [19, 20]. When the expectancy characteristic is the larger the better or the smaller the better, the normalized value of Grey relation can be described as formula (5) and (6), respectively:

5 $$ {x}_i^{\ast }(k)=\frac{x_i^0(k)-\min \left\{{x}_i^0(k)\right\}}{\max \left\{{x}_i^0(k)\right\}-\min \left\{{x}_i^0(k)\right\}}\kern1em \left(i=1\sim m,k=1\sim n\right) $$

6 $$ {x}_i^{\ast }(k)=\frac{\max \left\{{x}_i^0(k)\right\}-{x}_i^0(k)}{\max \left\{{x}_i^0(k)\right\}-\min \left\{{x}_i^0(k)\right\}}\kern1em \left(i=1\sim m,k=1\sim n\right) $$

where ${x}_i^{\ast }(k)$ denotes the *i*th value’s normalized value from the kth data series, ${x}_i^0(k)$ represents the *i*th value of initial result from the kth data series, *m* signifies the quantity of element from the data series, and *n* denotes the quantity of data series.

Grey relational coefficient for the data series expresses the connection between the perfect and practical data arrays. The Grey relational coefficient is defined as:

7 $$ \gamma \left({x}_0^{\ast }(k),{x}_i^{\ast }(k)\right)=\frac{\Delta_{\mathrm{min}}+\xi {\Delta}_{\mathrm{max}}}{\Delta_{0i}(k)+\xi {\Delta}_{\mathrm{max}}}\kern1em \left(i=1\sim m,k=1\sim n\right) $$

here

8 $$ {\Delta}_{0i}(k)=\left|{x}_i^{\ast }(k)-{x}_0^{\ast }(k)\right| $$

9 $$ {\Delta}_{\mathrm{min}}=\underset{\forall i}{\min}\underset{\forall k}{\min }{\Delta}_{0i}(k) $$

10 $$ {\Delta}_{\mathrm{max}}=\underset{\forall i}{\max}\underset{\forall k}{\max }{\Delta}_{0i}(k) $$

where ${x}_0^{\ast }(k)$ is the reference series and ${x}_i^{\ast }(k)$ is the comparability series; *ξ* is the distinguishing coefficient, *ξ* ∈ [0, 1], usually *ξ* = 0.5; furthermore, ${\Delta}_{\mathrm{min}}=\underset{\forall i}{\min}\underset{\forall k}{\min }{\Delta}_{0i}(k)$, ${\Delta}_{\mathrm{max}}=\underset{\forall i}{\max}\underset{\forall k}{\max }{\Delta}_{0i}(k)$.

The Grey relational grade reveals the degree of interrelation between the reference sequences and comparability series. Therefore, a greater value of GRG tends to show the relevant parameter combination is nearer to the optimal parameters set. In fact, GRG is the weight sum of the Grey relational coefficients. Then, the GRG can be calculated as:

11 $$ \gamma \left({x}_0^{\ast },{x}_i^{\ast}\right)=\sum \limits_{k=1}^n{\beta}_k\gamma \left({x}_0^{\ast }(k),{x}_i^{\ast }(k)\right)\kern1em \left(k=1\sim n\right) $$

where $\gamma \left({x}_0^{\ast },{x}_i^{\ast}\right)$ denotes the GRG; *β*_*k*_ is the weight value of the kth response variable which can be obtained from the principal component analysis.

Grey relational grade is a significant way to reduce two optimal objectives to one. In addition, the GRG indicates the degree of influence of parameter levels on the quality characteristics. In the multi-objective optimization process, the experiment and measurement results in Table 3 were normalized at first. Subsequently, the deviation sequence *∆*_*0i*_ were calculated with the help of formula (8). And then, the Grey relational coefficient was obtained by means of formula (7). The results of deviation sequence and Grey relational coefficient are shown in Table 3. According to the principal component analysis, *β*_*1*_ = 87.306% and *β*_*2*_ = 12.694%; here, *β*_*1*_ and *β*_*2*_ are the weight value of the tensile strength and void content, respectively. So, the tensile strength is the first principal component, followed by void content. Then, the results of GRG were calculated on the basis of formula (11) as shown in Table 3. From the table, we can see the maximum and minimum values of the GRG are 0.9686 and 0.3337, respectively. It means that the combination with the heating temperature of 75 °C, tape tension of 300 N, roller pressure of 1500 N, and winding velocity of 10 rpm are the best parameters combination for the composite winding process. Meanwhile, the heating temperature of 50 °C, tape tension of 300 N, roller pressure of 1500 N, and winding velocity of 15 rpm are the worst parameters combination.

**Table 3:** Grey relational grade results

No.	Deviation sequence ∆_*0i*_		Grey relational coefficient		GRG
	Tensile strength	Void content	Tensile strength	Void content	
1	0.6915	0.8976	0.4196	0.3578	0.4116
2	0.5790	0.7840	0.4634	0.3894	0.4538
3	0.4162	0.2316	0.5457	0.6835	0.5635
4	0.5575	0.8043	0.4728	0.3834	0.4613
5	0.0247	0.0000	0.9529	1.0000	0.9590
6	0.1789	0.3507	0.7365	0.5878	0.7173
7	0.3064	0.6110	0.6201	0.4500	0.5981
8	0.6322	0.8540	0.4416	0.3693	0.4323
9	0.0192	0.0697	0.9630	0.8777	0.9520
10	0.0312	0.0950	0.9412	0.8403	0.9282
11	0.3785	0.8390	0.5691	0.3734	0.5439
12	0.5200	0.7043	0.4902	0.4152	0.4805
13	0.4016	0.6012	0.5546	0.4541	0.5416
14	0.5565	0.6731	0.4732	0.4262	0.4672
15	0.7699	0.5356	0.3937	0.4828	0.4052
16	0.0169	0.0392	0.9673	0.9272	0.9622
17	0.0000	0.1607	1.0000	0.7568	0.9686
18	0.1651	0.2717	0.7518	0.6479	0.7384
19	0.8868	0.6390	0.3606	0.4390	0.3707
20	0.7435	0.3798	0.4021	0.5683	0.4236
21	0.8234	0.9061	0.3778	0.3556	0.3749
22	0.1933	0.3077	0.7212	0.6190	0.7080
23	1.0000	0.9885	0.3333	0.3359	0.3337
24	0.6559	0.7750	0.4326	0.3922	0.4273
25	0.1560	0.2588	0.7622	0.6589	0.7488
26	0.4488	0.7431	0.5270	0.4022	0.5109
27	0.3261	0.6110	0.6053	0.4500	0.5852
28	0.6303	1.0000	0.4424	0.3333	0.4283
29	0.2134	0.6390	0.7008	0.4390	0.6670

### Modeling of Backpropagation Neural Network

The backpropagation neural network generally includes three layer constructions which are input layer, hidden layer, and output layer. In order to obtain better results, a supervised learning algorithm needs to be employed to train the neural networks. Later on, the object minimizing the summation of the mean square error for all output layers is used to regulate the weights and thresholds of before and after layer [21, 22]. Particularly, during the training process, the neural network’s weights and thresholds are amended in a manner of repeated iteration until the trained network shows good agreement with the training sets [23]. Meanwhile, the final configuration of backpropagation neural network will be concluded by the input series and output sequences. In this study, the tape winding process contains four process parameters, namely heating temperature, tape tension, roller pressure, and winding velocity. Hence, the variable of input layer including four parameters is set to 4. The output layer has only one neuron that is the calculated GRG. Meanwhile, the hidden layer of BPNN has a major impact on the network model’s robustness. In the paper, the quantity of hidden layer is set to 6. Finally, the training process of BPNN can be described as follows:

#### Step 1

Count outputs of nodes in the hidden layer. According to the calculation principle of the neural network, the hidden layer and the output layer can be respectively described as:

12 $$ {y}_j=f\left(\sum \limits_{i=1}^n{w}_{ij}{x}_i+{\theta}_j\right)\kern1em \left(i=1,2,\dots, n;\kern0.5em j=1,2,\dots, l\right) $$

13 $$ {z}_k=g\left(\sum \limits_{j=1}^m{v}_{jk}f\left(\sum \limits_{i=1}^n{w}_{ij}{x}_i+{\theta}_j\right)+{\eta}_k\right) $$

where *x*_*i*_ denotes the *i*th input variable in the input layer, *y*_*j*_ denotes the *j*th neuron’s output from the hidden layer, and *z*_*k*_ denotes the *k*th neuron’s output from the output layer; *w*_*ij*_ denotes the connection weighs between neuron *i* in the input layer and neuron *j* in the hidden layer; *θ*_*i*_ is the threshold value of neuron *j* in the hidden layer; *v*_*jk*_ denotes the connection weighs between neuron *j* in the hidden layer and the neuron *k* in the output layer; and *η*_*k*_ is the threshold value of neuron *k* in the output layer*.* In addition, *f(x)* denotes the transfer function between input and hidden layer; here, we choose the Sigmoid function, and *g(x)* is the transfer function between the hidden layer and output layer; here, we select the Purelin function just as shown in formula (14).

14 $$ \left\{\begin{array}{l}f(x)=\frac{1}{1+{e}^{-\alpha x}}\\ {}g(x)= ax+b\end{array}\right. $$

where *α*, *a*, and *b* are all the coefficient of the model. For simplicity, we can use *α* = *a = b* = 1.

#### Step 2

Count the output data of BPNN. Let *z* denote the final output of the model, then the backpropagation neural network model can be described as follow:

15 $$ z=\sum \limits_{j=1}^6\frac{\nu_j}{1+\exp \left(-\sum \limits_{i=1}^4{w}_{ij}{x}_i-{\theta}_j\right)}+\eta $$

#### Step 3

Minimize the mean square error. To improve and refine the accuracy of the BPNN model, the mean square error function between the actual and trained outputs should be defined, that is,

16 $$ E=\frac{1}{2}\sum \limits_{k=1}^m{\left({z}_k-{R}_k\right)}^2 $$

where *E* is the mean square error; *R*_*k*_ denotes the desired output.

Since we had obtained the GRG, the BPNN was applied to establish the non-linear mapping relationship between process parameters and the GRG. In this step, the BPNN was composed of three-layer networks including one input layer with four neurons, one hidden layer with six neurons, and one output layer with one neuron. For the composite tape winding process, the foremost parameters heating temperature *T*, tape tension *F*, roller pressure *P*, and winding velocity *V* were selected as the input neurons. The GRG was the only one output neuron and also the ultimate optimal goal of the neural network model.

**Fig. 5:**
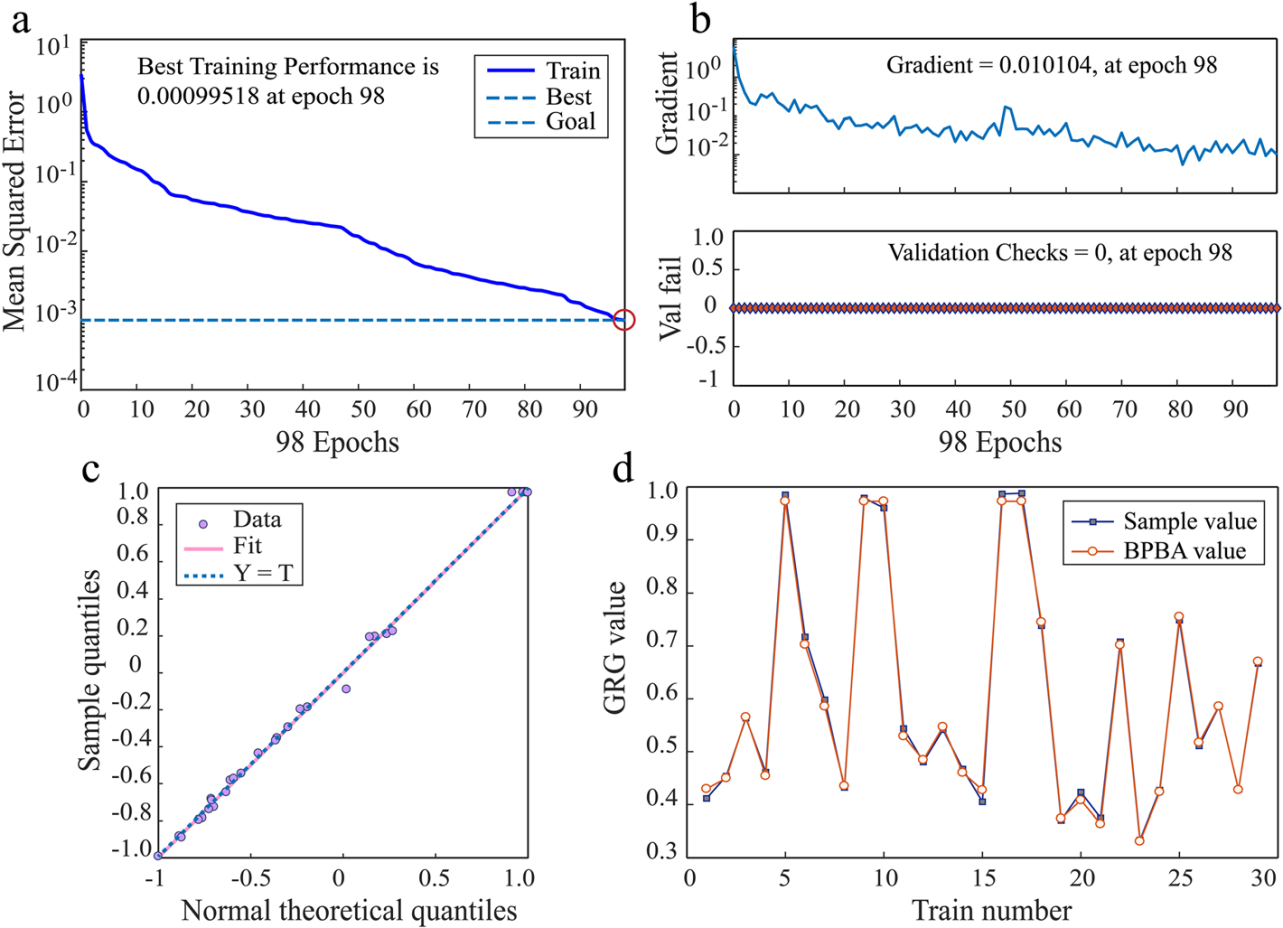
The training process and results of backpropagation neural network. **a** The error curve of GRG in BP network training process. **b** Error curve of training process. **c** Normal quantile-quantile plot. **d** Comparison of measured value and prediction value

The modeling process for BPNN was carried out via the MATLAB version R2017a. The sample data coming from Table 4 were applied to train the neural network model. When the expected error precision was achieved, the training process stopped. Figure 5 shows the training process and results of backpropagation neural network. According to Fig. 5a and b, we can see the best training performance is 0.00099518 at the 98th training times. In Fig. 5c, the points fall on the reference line which means the two data sets come from the normal distribution. Meanwhile, the prediction value is closely matched with the measured results as shown in Fig. 5d. However, to further verify the prediction accuracy of the model, some additional validation experiments should be put into effect. Three tests were selected randomly from the process parameters interval of tape winding manufacturing. As can be seen in Table 4, the error between the experimental value and simulated value is less than 6.5%. In a word, the validation test shows a high fitting accuracy for the trained model.

**Table 4:** Experimental verification of BP neural network model

No	*T*	*F*	*P*	*V*	Experiment results		GRG value		Error (%)
					Tensile strength (MPa)	Void content (%)	Simulation	Experiment	
1	91	325	659	7.8	972.83	0.59	0.62854	0.67143	6.39
2	73	60	1327	5.6	1088.42	0.41	0.57304	0.54732	4.49

**Fig. 6:**
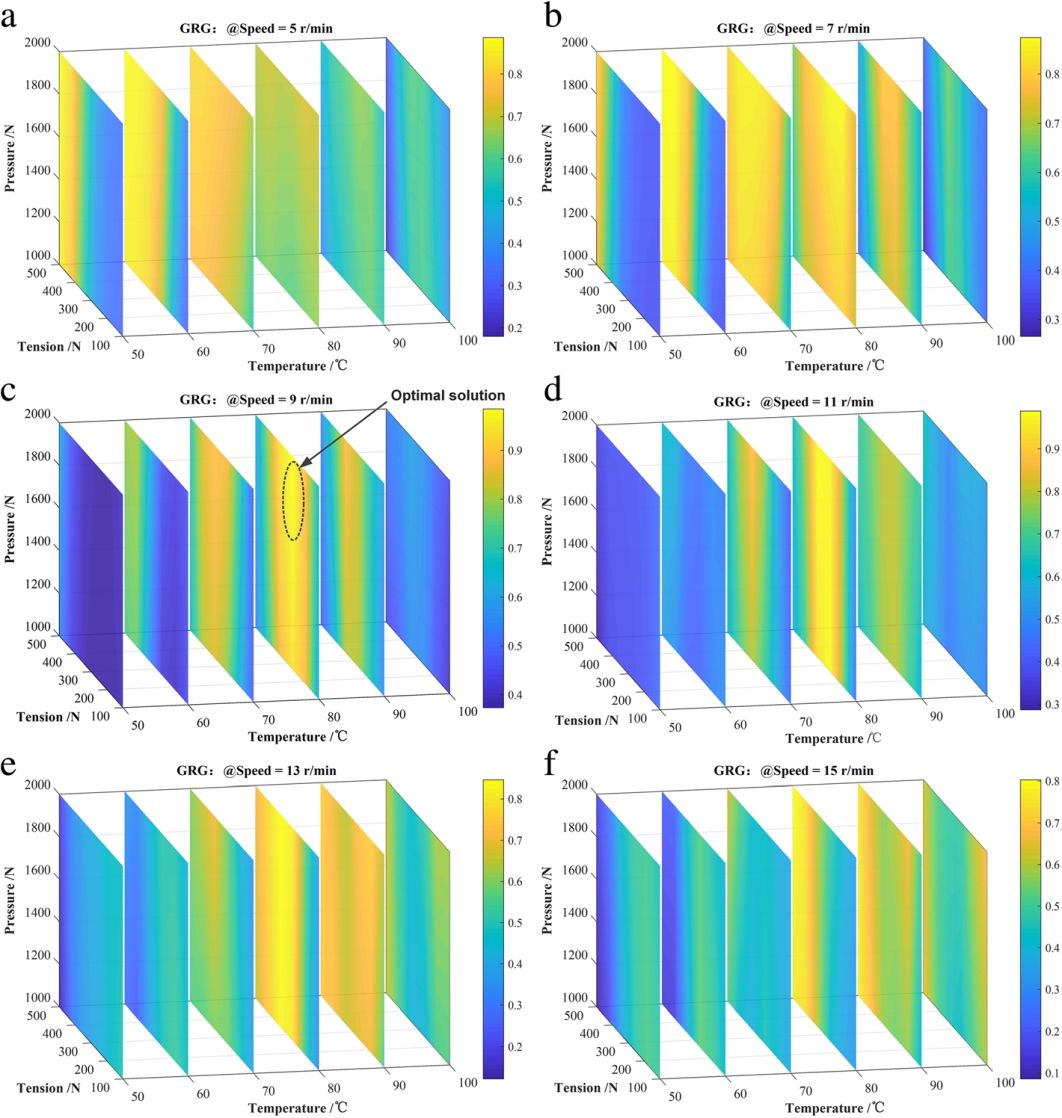
The slice in 3D view for process parameters influence on the GRG. **a** Speed = 5 r/min, parameters influence on GRG. **b** Speed = 7 r/min, parameters influence on GRG. **c** Speed = 9 r/min, parameters influence on GRG. **d** Speed = 11 r/min, parameters influence on GRG. **e** Speed = 13 r/min, parameters influence on GRG. **f** Speed = 15 r/min, parameters influence on GRG

**Fig. 7:**
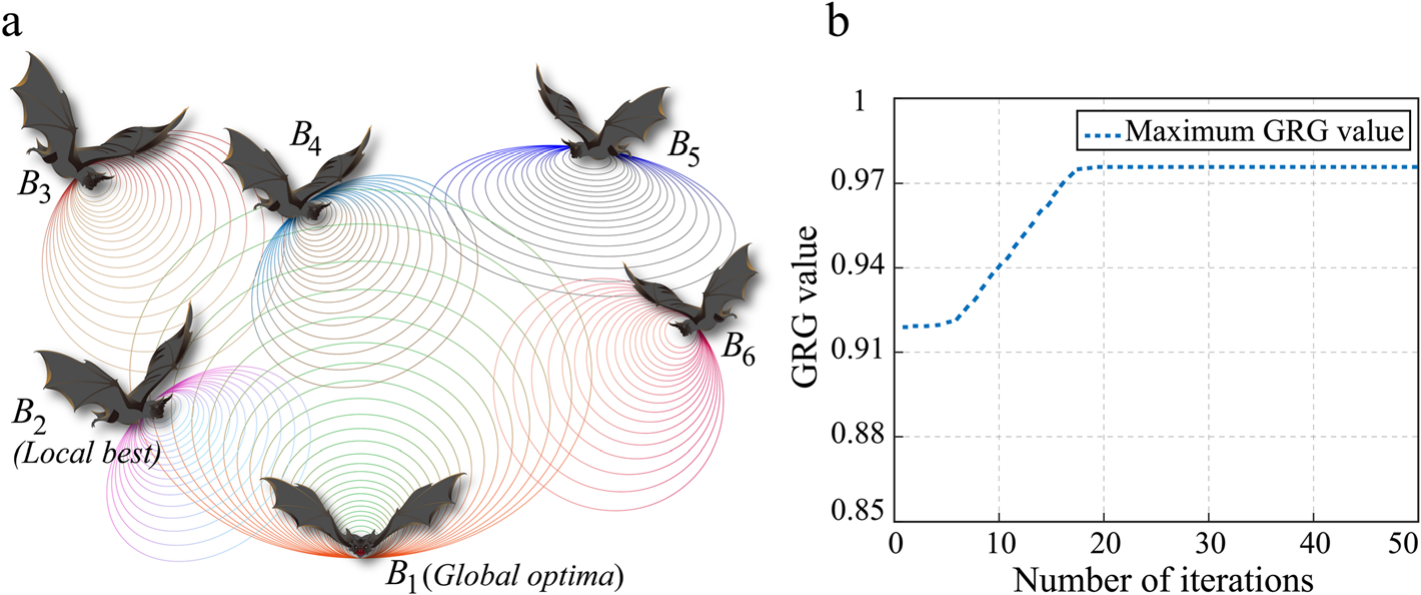
Bat-inspired optimization algorithm. **a** The schematic diagram of bat algorithm. **b** Iteration process with bat algorithm

In order to describe the GRG changing with the process parameters more intuitively, the response contour for the GRG with respect to process parameters based on the BP neural network model is drawn in Fig. 6. Each slice in 3D view consists of different color values which express the corresponding Grey relational grade value. The corresponding color bar on the right side of each thumbnail in Fig. 7 shows the congruent relationship between color and GRG value. According to the slice planes, it is quite clear that the GRGs have no evident changes with the roller pressure increasing or decreasing in most situations. The GRG increases with the increase of the roller pressure only at the high winding velocity and heating temperature. The reason could be that the roller pressure has a great impact on the void content of winding products in the composite tape winding process. However, the void content has a lower proportion in the GRG according to the principal component analysis. When the heating temperature is low, the GRG decreases as the tape tension rises. Meanwhile, in the higher tension range, the decreasing trend of GRG as the increasing of tape tension is more obvious than the other parameter range. At high temperature, the GRG increase at the early stage and then decrease with the increasing of tape tension. In the composite tape winding process, winding velocity has an obvious influence on the performances of winding products. The GRG has a higher value at the lower winding velocity. Looking at the slice planes as a whole, the high peak values of GRG are located in the condition of medium temperature, low velocity, and high tension. In general, void content is more likely to be affected by roller pressure, and tensile strength is more likely to be influenced by tape tension.

### Optimal Grey Relational Grade via Bat Algorithm

The bat algorithm is a patent evolutionary algorithm inspired by microbats’ echolocation behavior when looking for preys or avoiding barriers in a condition of complete darkness [24]. Actually, the microbat transmits the quite loud acoustic pulses and concurrently listens to the echoes bouncing off the ambient objects, so that the microbats can be able to develop the hunting strategy for prey taking full advantage of the sound pulses properties [25]. Figure 7a describes the hunting process of microbats. From the picture, we can see each bat has an individual location and emits a sound pulse which has a steady loudness and frequency domain so as to amend the location and velocity in real time.

At first, the microbat has a position *x*_*i*_ and velocity *v*_*i*_ in the multi-dimensional predatory searching space. Then, the updated position ${x}_i^t$ and velocity ${v}_i^t$ with a time step *t* can be provided by

17 $$ {f}_i={f}_{\mathrm{min}}+\left({f}_{\mathrm{max}}-{f}_{\mathrm{min}}\right)\beta $$

18 $$ {v}_i^t={v}_i^{t-1}+\left({x}_i^t-{x}_{\ast}\right){f}_i $$

19 $$ {x}_i^t={x}_i^{t-1}+{v}_i^t $$

where *β* ∈ [0, 1] denotes a random vector extracted from the uniform distribution; *x*_∗_ represents the latest global optimal position selecting from all the *n* bats’ solutions; *f*_*i*_ is the frequency of bat *i* and *λ*_*i*_ is the wavelength of bat *i*; *f*_*i*_ (or *λ*_*i*_) is usually applied to modify the velocity changing while fixing the other factor *λ*_*i*_ (or *f*_*i*_). In general, we use *f*_min_ = 0 and *f*_max_ = 100. Originally, each bat has a random frequency extracted from [*f*_min_, *f*_max_]. When a solution has been produced from the latest optimal solutions, a new resolve project for each bat will be produced as follow:

20 $$ {\mathrm{x}}_{\mathrm{new}}={\mathrm{x}}_{\mathrm{old}}+\varepsilon {A}^t $$

where *ε* ∈ [0, 1] denotes a random number; ${A}^t=<{A}_i^t>$ denotes the average loudness of all the bats at time step *t*.

With iterations going on, the loudness *A*_*i*_ and pulse emission rate *r*_*i*_ will be renewed synchronously. Naturally, once a bat has found the prey, the sound loudness will generally decrease. At the same time, the pulse emission rate will increase, then the loudness can be set as any random value. For simplicity’s sake, we choose *A*_*0*_ = 1 and *A*_min_ = 0, and assuming *A*_min_ = 0 means that a bat sends no sound provisionally as the reason for finding a prey. Then, we can get

21 $$ {A}_i^{t+1}=\sigma {A}_i^t $$

22 $$ {r}_i^{t+1}={r}_i^{t+1}\left[1-\exp \left(-\gamma t\right)\right] $$

where *σ* and *γ* both are constants. In practice, *σ* is analogous to the cooling factor of the simulated annealing. For simplicity, we can also use *σ* = *γ* = 0.9 in the simulation.

Based on the Grey relational analysis, BP neural networks model for the tape winding process had been established subsequently. Then, the bat algorithm was applied to search the optimal parameters combination for the GRG. Compared to other algorithms, bat algorithm has more advantages such as high seeking velocity and efficaciousness. The formulation of the process optimization problem for producing maximum allowable GRG can be expressed as follows:

23 $$ {\displaystyle \begin{array}{l}\mathrm{Find}:x=\left(T,F,P,V\right)\\ {}\operatorname{Maximize}: GRG= BPNN(x)\\ {}\mathrm{Subject}\ \mathrm{to}:\left\{\begin{array}{l}50\kern0.5em {{}^{\circ}\mathrm{C}}\le T\le 100\kern0.5em {{}^{\circ}\mathrm{C}}\\ {}100N\le F\le 500N\\ {}1000N\le P\le 2000N\\ {}5\kern0.5em rpm\le V\le 15\kern0.5em rpm\end{array}\right.\end{array}} $$

where *T* is the heating temperature, °C; *F* is the tape tension, *N*; *P* is the roller force, *N*; and *V* is winding speed, rpm.

The optimization process is performed on the MATLAB version R2017a software platform. Figure 7b shows the iteration process of bat-inspired optimization algorithm. It is obvious that the convergence was achieved within 20 iterations. According to the optimization results, the optimal parameter combination is heating temperature with 73.8 °C, tape tension with 291.2 N, roller pressure with 1804.1 N, and winding velocity with 9.1 rpm. And the highest GRG is 0.9829. The corresponding desirability value is 0.9504 which demonstrates the high agreement between the target data and the objective value. Moreover, the optimal parameter combination conforms to the optimized process parameter intervals based on the sensitivity analysis which is published on the authors’ another article [7]. From that paper, the optimized intervals of the process parameters are as follows: heating temperature within 62.5 and 75 °C, tape tension within 200 and 300 N, roller pressure within 1500 and 2000 N, and winding velocity within 5 and 10 rpm. Therefore, this paper testifies the necessity and availability of “the optimal parameter interval for composite tape winding process” on the other side.

### Verification

To validate the optimal parameter combination, a confirmation experiment was carried out. Firstly, the highest GRG value 0.9686 in the 17th experiment, namely heating temperature of 75 °C, tape tension of 300 N, roller pressure of 1500 N, and winding velocity of 10 rpm, was selected as the initial process condition setting. Then, the comparison between the initial experiment and optimal settings was accomplished as shown in Table 5. The corresponding desirability value is 0.9796 which demonstrates the high agreement between the target data and the objective value. The value of tensile strength increased from 1215.31 to 1329.62 MPa. Meanwhile, the value of void content decreased from 0.15 to 0.137%. It is observed that the changing of the void content had a minor impact on the GRG of the research model. Nevertheless, it is undeniable that the void has a non-negligible effect on the properties of the winding product. The results make it clear that the proposed method in this paper can be utilized to guide the composite tape winding manufacturing process. The optimal parameter settings can improve the tensile strength and reduce the void content for the composite tape winding products. And the performance of winding products can be improved to a certain degree.

**Table 5:** Comparison between the initial and optimal settings

	*T*	*F*	*P*	*V*	Experiment results		GRG
					Tensile strength (MPa)	Void content (%)	
Initial	75	300	1500	10	1215.31	0.15	0.9686
Optimal	73.8	291.2	1804.1	9.1	1329.62	0.137	0.9796
Improvement					+ 15.69	− 0.013	+ 0.0110

## Conclusions

In this paper, an integrated methodology combining Grey relational analysis, BP neural network, and bat algorithm was established to obtain the optimal technology parameters of composite tape winding process. In the composite tape winding process, processing parameters including heating temperature, tape tension, roller pressure, and winding velocity play a crucial role. A satisfied composite tape winding technological parameter combination aiming at two different objectives, including physical performance, mechanical property, or others, would actively guide tape winding process. Therefore, preferable winding parameters combination will improve both physical and mechanical performance for the composite winding productions. In this paper, void content and tension strength were selected as the two performance indexes for winding products. At first, the Grey relational analysis successfully transformed the multi-response problem into a single-objective optimization problem. According to the principal component analysis, the weight value of the tensile strength was much larger than the void content which means the tensile strength occupies a more important proportion in the GRG. BP neural networks were applied to establish the nonlinear mapping relations between the process parameters and GRG. Due to the high seeking velocity and efficaciousness, bat algorithm was applied to search the optimal parameters combination for the GRG in the last step.

According to the optimization results, the optimal parameter combination is heating temperature with 73.8 °C, tape tension with 291.2 N, roller pressure with 1804.1 N, and winding velocity with 9.1 rpm. And the corresponding tensile strength increased by 15.69 MPa and void content decreased by 0.013%. It is observed that the changing of the void content had a minor impact on the GRG of the research model. Nevertheless, it is undeniable that the void has a non-negligible effect on the properties of the winding product. The verification test validated that the optimized intervals of the process parameters were reliable and stable for winding products manufacturing. The optimal parameter settings can improve the tensile strength and reduce the void content for the composite tape winding products. And the performance of winding products can be improved to a certain degree. At last, the developed method GRA-BPNN-BA was verified to be useful for the multi-objective optimization problem in the manufacturing industry.

## Data Availability

The datasets supporting the conclusions of this article are included within the article.

## Abbreviations

BA: Bat algorithm
BBD: Box–Behnken design
BPNN: Backpropagation neural network
GRA: Grey relational analysis
GRG: Grey relational grade
